# Polymorphisms in *HSD17B1*: Early Onset and Increased Risk of Alzheimer's Disease in Women with Down Syndrome

**DOI:** 10.1155/2012/361218

**Published:** 2012-03-04

**Authors:** Joseph H. Lee, Susan Gurney, Deborah Pang, Alexis Temkin, Naeun Park, Sarah C. Janicki, Warren B. Zigman, Wayne Silverman, Benjamin Tycko, Nicole Schupf

**Affiliations:** ^1^The Taub Institute for Research on Alzheimer's Disease and the Aging Brain, Columbia University Medical Center, New York, NY 10032, USA; ^2^Gertrude H. Sergievsky Center, Columbia University Medical Center, New York, NY 10032, USA; ^3^Department of Epidemiology, Columbia University Medical Center, New York, NY 10032, USA; ^4^Department of Psychology, New York State Institute for Basic Research in Developmental Disabilities, Staten Island, NY 10314, USA; ^5^Department of Pathology, Columbia University Medical Center, New York, NY 10032, USA; ^6^Department of Neurology, Columbia University Medical Center, New York, NY 10032, USA; ^7^Kennedy Krieger Institute and Johns Hopkins University School of Medicine, Baltimore, MD 21205, USA; ^8^Department of Psychiatry, Columbia University Medical Center, New York, NY 10032, USA

## Abstract

*Background/Aims*. Genetic variants that affect estrogen activity may influence the risk of Alzheimer's disease (AD). In women with Down syndrome, we examined the relation of polymorphisms in hydroxysteroid-17beta-dehydrogenase (*HSD17B1*) to age at onset and risk of AD. *HSD17B1* encodes the enzyme 17*β*-hydroxysteroid dehydrogenase (HSD1), which catalyzes the conversion of estrone to estradiol. *Methods*. Two hundred and thirty-eight women with DS, nondemented at baseline, 31–78 years of age, were followed at 14–18-month intervals for 4.5 years. Women were genotyped for 5 haplotype-tagging single-nucleotide polymorphisms (SNPs) in the *HSD17B1* gene region, and their association with incident AD was examined. *Results*. Age at onset was earlier, and risk of AD was elevated from two- to threefold among women homozygous for the minor allele at 3 SNPs in intron 4 (rs676387), exon 6 (rs605059), and exon 4 in *COASY *(rs598126). Carriers of the haplotype TCC, based on the risk alleles for these three SNPs, had an almost twofold increased risk of developing AD (hazard ratio = 1.8, 95% CI, 1.1–3.1). *Conclusion*. These findings support experimental and clinical studies of the neuroprotective role of estrogen.

## 1. Introduction

The neurotrophic and neuroprotective mechanisms of estrogen have beneficial effects on brain function that include increases in cholinergic activity [1–5], antioxidant activity [6, 7], and protection against the neurotoxic effects of beta amyloid [8–11]. Thus, the dramatic declines in estrogen following menopause may contribute to higher risk of AD in women [12]. 

Allelic variation in genes within the estrogen biosynthesis and estrogen receptor pathways may modify cerebral estrogen activity and influence risk of AD. The hydroxysteroid-17beta-dehydrogenase (*HSD17B1*) gene, located on chromosome 17q11-q21, encodes the enzyme 17*β*-hydroxysteroid dehydrogenase (HSD1), which catalyzes the conversion of estrone to estradiol. Variants in *HSD17B1* have been examined for their relation to hormone levels, [13, 14] breast cancer [13, 15–24], endometriosis and endometrial cancer [13, 25–29], colorectal cancer [30, 31], and prostate cancer [32], with inconsistent results. Studies of polymorphisms in *HSD17B1* have focused on rs605059, a nonsynonymous single-nucleotide polymorphism in exon 6. The T/C polymorphism in rs605059, the change in bases at codon 313 in exon 6, is expressed as the change in amino acids from serine to glycine. The rs605059 SER313GLY variant has been associated with a modestly increased risk of endometriosis, estrogen receptor-negative tumors in breast cancer patients, and colorectal cancer in women, conditions known to be associated with estrogen regulation [16, 28, 29, 31], but not all studies have found positive associations. Along with two other haplotype-tagged SNPs, (rs676387 and rs598126), these common variants represent over 80% of the variation at this locus. [13]. Expression of *HSD17B1* was found to be increased in prefrontal cortex in late-stage AD [33], but variants in *HSD17B1 *have not been examined for their association with age at onset or risk of AD. 

 Women with Down syndrome (DS) are at high risk for AD, with the onset of dementia 10–20 years earlier than women in the general population [34–36]. Early age at menopause and low levels of bioavailable estradiol in postmenopausal women with DS are both associated with earlier onset and increased cumulative incidence of AD [37, 38], suggesting that the decline in estrogen contributes to pathological processes leading to AD in this high-risk population. In this study, we examined the relationships between single-nucleotide polymorphisms in *HSD17B1*, age at onset, and cumulative incidence of AD in women with DS to determine if genotype was related to risk. 

## 2. Materials and Methods

### 2.1. Subjects

The initial cohort included a community-based sample of 279 women with DS. Of these 279 women, 252 (90.3%) agreed to provide a blood sample, and 244 (96.8%) were genotyped for *HSD17B1*. All individuals were 30 years of age or older at study onset and resided in New York, New Jersey, Pennsylvania, or Connecticut. In all cases, a family member or correspondent provided informed consent, including blood sampling and genotyping, with participants providing assent. The distribution of level of intellectual disability and residential placement did not differ between participants and those who did not participate. Recruitment, informed consent, and study procedures were approved by the Institutional Review Boards of Columbia University Medical Center and the New York State Institute for Basic Research in Developmental Disabilities.

### 2.2. Clinical Assessment

Assessments were repeated at 14–18-month intervals over five cycles of data collection and included evaluations of cognition and functional abilities, behavioral/psychiatric conditions, and health status. Cognitive function was evaluated with a test battery designed for use with individuals with DS varying widely in their levels of intellectual functioning, as described previously [39]. Structured interviews were conducted with caregivers to collect information on changes in cognition, function, and adaptive behavior. Past and current medical records were reviewed for all participants using a standardized protocol. 

### 2.3. Classification of Dementia

This is a longitudinal cohort study of onset of AD in women with Down syndrome. The classification of dementia status, dementia subtype, and age at onset was determined during consensus case conferences where information from all available sources was reviewed. Classifications were made blind to *HSD17B1* genotype. We classified participants into two groups, following the recommendations of the AAMR-IASSID Working Group for the Establishment of Criteria for the Diagnosis of Dementia in Individuals with Developmental Disability [40]. Participants were classified as nondemented if they were without cognitive or functional decline, or if they showed some cognitive and/or functional decline that was not of significant magnitude to meet dementia criteria (*n* = 164). Participants were classified as demented if they showed substantial and consistent decline over the course of follow up for at least one-year duration and had no other medical or psychiatric conditions that might mimic dementia (*n* = 80). Age at meeting criteria for dementia was used to estimate age at the onset of dementia. Of the 80 participants with dementia, three had a history of stroke or TIA and were excluded from the analyses. Three additional participants were also excluded because their findings suggestive of dementia may have been caused by another non-AD medical or psychiatric condition, leaving 164 nondemented and 74 demented women in the analysis. Only women with probable or possible AD were included in the dementia group for analysis.

### 2.4. DNA Isolation and Genotyping

Genomic DNA was extracted from peripheral blood leukocytes using the FlexiGene DNA kit (Qiagen). Isolation of DNA and genotyping were performed blind to the dementia status of the participant. We analyzed 4 single-nucleotide polymorphisms (SNPs) in *HSD17B1 *and one flanking SNP (rs598126) in CoA synthase (COASY), which is in high-linkage disequilibrium with rs605059. These included rs605059 (SER313GLY, C > T), which has been the SNP most consistently and strongly associated with estrogen-related disorders. Additional tagging SNPS were selected to provide coverage of the gene or to include SNPs which had also been associated with estrogen-related disorders in at least one study. These included rs2830 (T > C), rs2676530 (G > A), rs676387 (G > T), and rs598126 (C > T).  Table 2 provides the locations and allele frequencies of these SNPs. SNPs were genotyped using TaqMan PCR assays (Applied Biosystems) with PCR cycling conditions recommended by the manufacturer, and by Prevention Genetics using proprietary array tape technology. Accuracy of the genotyping (≥97%) was verified by including duplicate DNA samples by comparing the TaqMan and array tape data with results of restriction digestion polymorphisms (RFLPs) for several of the SNPs, and by testing for Hardy-Weinberg equilibrium. Not all genotypes were available for all women at all SNPs, so the numbers examined vary slightly by SNP.

### 2.5. Apolipoprotein E Genotypes

*APOE* genotyping was carried out by PCR/RFLP analysis using *Hha*I (*Cfo*I) digestion of an *APOE* genomic PCR product spanning the polymorphic (cys/arg) sites at codons 112 and 158, followed by acrylamide gel electrophoresis to document the restriction fragment sizes [41]. Participants were classified according to the presence or absence of at least one *APOE ε*4 allele. 

### 2.6. Potential Confounders

Potential confounders included level of intellectual disability, body mass index (BMI), ethnicity, and the presence of an *APOE ε*4 allele. Level of intellectual disability was classified as mild to moderate (IQ from 35 to 70) or severe to profound (IQ < 34), based on IQ scores obtained before the onset of AD. BMI was calculated as weight in kilograms divided by the squared height in square meters (kg/m^2^) and was measured at each evaluation. The baseline measure of BMI was used in the analysis and was included as a continuous variable. Ethnicity was categorized as white or nonwhite.

### 2.7. Statistical Analysis

Prior to association analysis, we tested all SNPs for Hardy-Weinberg Equilibrium using the HAPLOVIEW program [42], and all were found to be in Hardy-Weinberg equilibrium. SNPs were analyzed with a dominant model in which participants homozygous for the common allele were used as the reference group, with the exception of rs605059 and rs598126. We coded the C allele at rs605059 as the high-risk allele since previous work had shown that women carrying the C allele at rs605059 had lower levels of estradiol and a lower estradiol/estrone ratio than women carrying the TT genotype [13]. We coded the C allele as the high-risk allele in rs598126 since previous work had shown the TT genotype to be associated with increased risk of breast cancer [15]. To code the remaining genotypes, we used common alleles for *HSD17B1 *SNPs for Hapmap whites at the NCBI SNP web site (http://www.ncbi.nlm.nih.gov/projects/SNP/). In preliminary analyses, the  *X* ^2^ test (or the Fisher's exact test when any cell had <5 subjects) was employed to assess the association between AD and SNP genotypes as well as other possible risk factors for AD including ethnicity, level of intellectual disability, and the presence of an *APOE ε*4 allele. Analysis of variance (ANOVA) was used to examine BMI and age by AD status. 

The analysis was structured as a longitudinal cohort study of the onset of AD. We used Cox proportional hazards modeling to assess the relationship between *HSD17B1* genotypes, age at onset, cumulative incidence, and the hazard ratio of AD, adjusting for ethnicity, BMI, level of intellectual disability and the presence of an *APOE ε*4 allele. The time to event variable was age at onset for participants who developed AD and age at last assessment for participants who remained nondemented throughout the follow-up period. Because a set of three contiguous SNPs that span ~10 kb-rs676387, rs605059, and rs598126 were significantly associated with AD, we performed a haplotype analysis to identify haplotype(s) that may harbor a susceptibility variant(s) as implemented in the PLINK program [43]. For nearly all individuals, we were able to identify the most likely haplotypes from the genotype data with a high degree of certainty (i.e., the posterior probability approaching 1.0 for 91% of the cohort with the rest exceeding probability >0.7). Subsequently, we used the estimated haplotype as a “superlocus” (analogous to a microsatellite marker) to perform Cox proportional hazards modeling. We restricted the analysis to individuals with a posterior probability of carrying the haplotype of 1.0.

## 3. Results

### 3.1. Demographic Characteristics

The mean age of participants at baseline was 49.4 years (range 31.5 to 78.1), and 88 percent of the cohort were white. The mean length of follow-up was 4.5 (SD ± 2.4) years. Table 1 presents the demographic characteristics of the participants according to AD status. Participants who developed AD over the follow-up period were significantly older at baseline than nondemented participants (54.2 versus 47.3 years) and were more likely to have severe or profound level of intellectual function (52.7% versus 40.9%), but did not differ in the distribution of ethnicity or the frequency of the *APOE ε*4 allele. Women who developed AD had a significantly lower BMI at baseline than women who remained nondemented over the follow-up period. The mean age at onset of AD was 55.7 ± 6.4 years. 

### 3.2. Analysis of SNPs in HSD17B1

**Figure 1:**
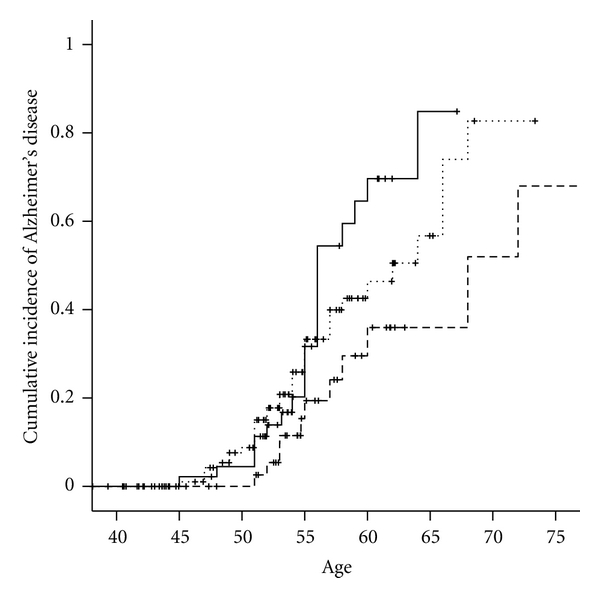
Cumulative incidence of Alzheimer's disease by *HSD17B1 *rs605059 genotype in women with Down syndrome. TT - - - -. CT……. CC—.

Table 2 shows the locations and minor allele frequencies (MAFs) of *HSD17B1 *SNPs for Hapmap whites at the NCBI SNP web site (http://www.ncbi.nlm.nih.gov/projects/SNP/) and for our cohort of women with DS. Allele frequencies were similar in women with DS to those observed in women without DS in the general population.  Table 3 presents the distributions of *HSD17B1 * genotypes and the association between *HSD17B1* SNPs and the hazard ratio of AD among women with Down syndrome, adjusted for age, ethnicity, level of intellectual disability, BMI, and the presence of an *APOE ε*4 allele. All of the 5 SNPs examined were in high-linkage disequilibrium (LD > 0.9, Figure 3). Three SNPs, rs676387, rs605059, and rs598126, showed significant associations with AD, the strongest being with rs605059. Women who carried one or two copies of the T allele at rs605059 were two to three times more likely to develop AD than women homozygous for the C allele (HR = 2.0, 95% CI, 0.98–4.2 for those with the CT genotype and HR = 3.0, 95% CI, 1.4–6.8 for those with the TT genotype) (Table 3) and had both earlier onset and higher cumulative incidence of AD over followup (Figure 1). The effects of carrying risk alleles for rs605059 were primarily seen in women over 60 years of age (Figure 1). 

**Figure 2:**
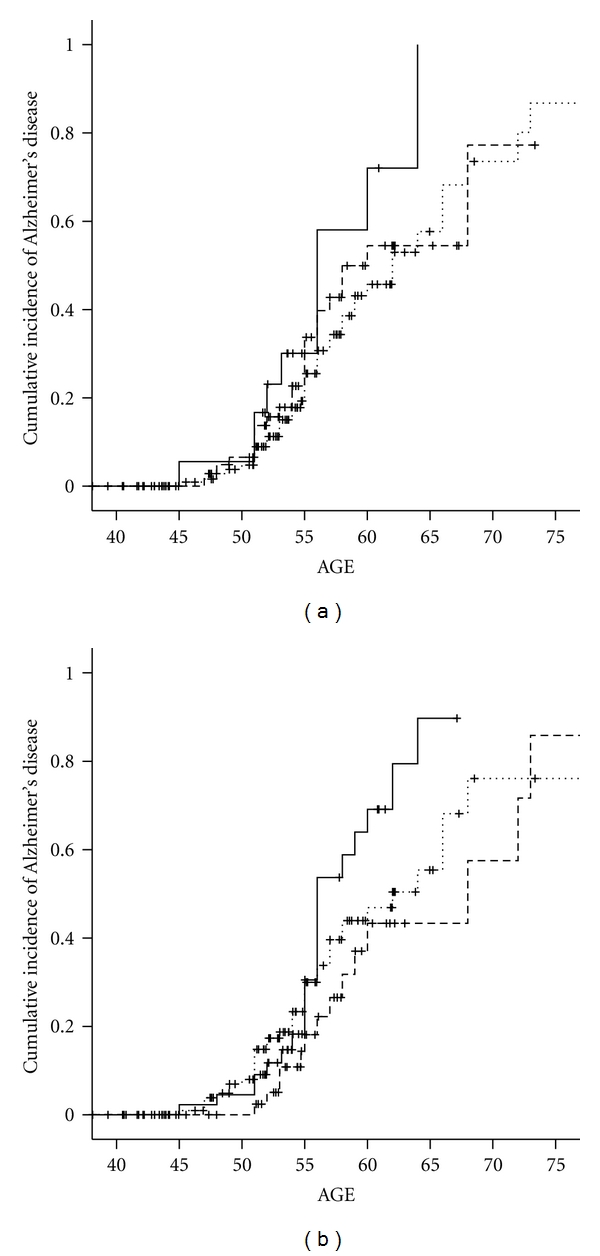
(a) Cumulative incidence of Alzheimer's disease by *HSD17B1 *rs598126 genotype in women with Down syndrome. TT - - - -. CT……. CC—. (b) Cumulative incidence of Alzheimer's disease by *HSD17B1 *rs676387 genotype in women with Down syndrome. CC - - - -. CT……. TT—.

**Figure 3:**
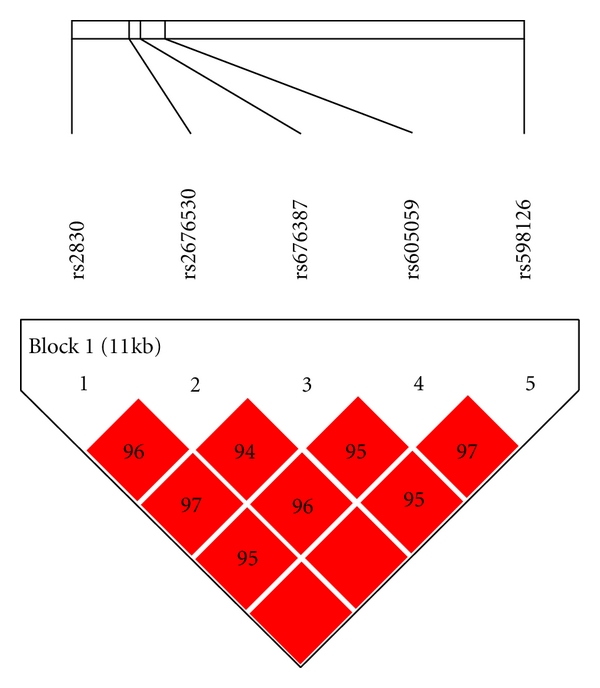
Linkage disequilibrium patterns for SNPs in *HSD17B1*.

The relation of rs676387 and rs598126 to increased risk for AD was seen only among women homozygous for the risk allele (Table 3) and was associated to a two- and one half-fold hazard ratio (HR_rs676387_ = 2.7, 95% CI: 1.2–5.8 and HR_rs5998126_ = 2.2, 95% CI: 1.1–4.4) and with earlier onset but not higher cumulative incidence of AD (Figures 2(a) and 2(b)).

### 3.3. Haplotype Analysis of the Three SNPs in a Cox Proportional Hazards Modeling

We first computed the most likely haplotypes for each individual and then used the haplotypes as a “super locus” to estimate hazard ratios controlling for potential confounders. Our haplotype analysis using rs676387-rs605059-rs598126 revealed that the carriers of haplotype TCC had earlier onset of AD, after adjusting for the presence of an *APOE ε*4, allele level of intellectual disability, ethnicity, and BMI (hazard ratio = 1.8, 95% CI, 1.1–3.1).

## 4. Discussion

Three of the five SNPs examined in *HSD17B1* were associated with increased risk of AD. Women who were heterozygous or homozygous for the C allele at rs605059 were two to three times as likely to develop AD as those carrying the TT genotype. Women with DS homozygous for the T allele at rs676387 or the C allele at rs598126 had 2.7 and 2.2-fold increased risk of AD, respectively, compared with women without these risk alleles, although risk was only slightly increased in women who were heterozygous for the risk allele. Carrying a high-risk allele at rs605059 was associated with both early onset and higher cumulative incidence, while carrying a high-risk allele at rs676387 or at rs598126 was associated primarily with earlier onset. Haplotype-based Cox proportional hazards model continued to support that TCC carriers had an almost 2-fold risk of developing AD after adjusting for covariates.

Polymorphisms or haplotypes in *HSD17B1* have been associated with increased risk for estrogen receptor-negative breast cancer, endometriosis, and endometrial cancer, but these associations have been modest and inconsistent [13, 15–32]. The *HSD17B1* gene encodes the enzyme HSD1 which catalyzes the conversion of estrone to estradiol. One pathway by which variants in *HSD17B1* could influence risk for AD is through changing the activity of HSD1 leading to changes in circulating estrogen levels. After menopause, the primary form of estrogen is estrone, which is formed in adipose tissue, muscle, liver, bone marrow, brain, and fibroblasts from aromatization of circulating androstenedione [44]. Increased body mass index in postmenopausal women is correlated with higher levels of serum estradiol and estrone [45, 46]. Low BMI has been found to be a risk factor for cognitive decline and risk for AD in late life [39, 47–49], and BMI may decline decades before onset of AD [50]. Among postmenopausal women not using hormone replacement therapy, nonobese women (<25 BMI) who were heterozygous or homozygous for the C allele at rs605059 had lower levels of estradiol and a lower estradiol/estrone ratio than women carrying the TT genotype [13], while no corresponding effects on estrone or estradiol levels were seen in women with BMI > 25. Our results showing earlier age at onset and higher cumulative incidence of AD among women carrying the C allele at rs605059 are consistent with this finding. Among non-obese women, variants in *HSD17B1* have also been associated with a more rapid rate of decline in estradiol levels during the perimenopausal period [51]. Low estrogen levels have been associated with increased risk of cognitive impairment and AD [38, 52–59], although some studies have found high levels of total estradiol in women with AD [60, 61]. A role for low estrogen in AD has also been supported by experiments in which estrogen deficiency accelerated amyloid plaque formation in transgenic mouse models of AD [62, 63]. The findings from this study are consistent with a role for *HSD17B1* in modifying risk of AD through influences on peripheral or central estrogen levels and point to the potential for hormonal replacement therapy to delay onset of AD in this high-risk population. 

*HSD17B1* belongs to the family of short-chain dehydrogenases/reductases (SDRs) of which at least 11 other 17-beta HSD types are under study, named for their sequence homology to *HSD17B1 *[64]. One of these, 17-beta HSD10, has demonstrated involvement with AD through binding with amyloid-beta [65]. While the substrate activity of *HSD17B1* is quite restricted, unlike that of 17beta HSD10, the multifunctionality of all SDRs is just beginning to be explored. For example, increased expression of *HSD17B1* and aromatase have been found in the prefrontal cortex of AD patients during the later stages of the disease [33]. It has been suggested that estradiol is upregulated in astroglia during AD, much as it is in reactive astroglia following brain injury, and increased expression of aromatase and *HSD17B1* may determine differences in levels of protective neurosteroids in the prefrontal cortex [33]. Continued work on genetic factors affecting neurosteroid activity may help to understand differences in rates of cognitive aging and risk of dementia. 

## Figures and Tables

**Table 1:** Demographic characteristics.

Characteristic	Nondemented	Alzheimer's disease
*N*	164	74
Age at baseline (M, SD)**	47.3 ± 6.9	54.2 ± 6.7
Level of intellectual disability (*n*, %)		
Mild/moderate	97 (59.1)	35 (47.3)
Severe/profound	67 (40.9)	39 (52.7)
Ethnicity (*n*, %)		
Non-hispanic white	142 (86.6)	68 (91.9)
Nonwhite	22 (13.4)	6 (8.1)
Body mass index (M, SD)**	29.9 ± 6.7	28.0 ± 6.0
Apolipoprotein E *ε*4 allele (*n*, %)	34 (21.0)	20 (27.0)

**P < 0.05.

**Table 2:** *HSD17B1* SNP chromosomal location^a^.

SNP	Chromosome position^a^	Distance from previous SNP	Minor allele	MAF^b^ observed	MAF from NCBI*	SNP location relative to *HSD17B1 *
rs2830	37958089		C	0.485	.392	Exon1
rs2676530	37959481	1392	A	0.230	.263	Intron 4
rs676387	37959799	318	T	0.259	.337	Intron 4
rs605059	37960432	633	T	0.482	.443	Exon 6
rs598126	37970046	9614	T	0.491	.429	Exon 4 of COASY

^a^Physical position on chromosome: Hg18, March 2006 assembly, dbSNP build 130.

^b^MAF: Minor allele frequency.

*http://www.ncbi.nlm.nih.gov/.

**Table 3:** Alzheimer's disease risk by HSD17B1 genotype in women with Down syndrome.

*HSD17B1 *genotype*	*N*	AD	HR (95% CI)**
rs2830			
CC	49	15 (30.6)	0.7 (0.3.5)
CT	101	31 (33.7)	0.9 (0.5–1.8)
TT	56	15 (26.8)	1.0 (reference)
rs2676530			
AA	14	7 (50.0)	1.5 (0.7–3.3)
AG	75	17 (22.7)	0.7 (0.4–1.2)
GG	135	47 (34.8)	1.0 (reference)
rs676387			
TT	22	9 (40.9)	**2.7 (1.2**–**5.8)**
GT	72	23 (31.9)	1.4 (0.8–2.4)
GG	129	40 (31.0)	1.0 (reference)
rs605059			
CC	59	20 (33.9)	**3.0 (1.4**–**6.8)**
CT	107	34 (31.8)	2.0 (0.98–4.2)
TT	51	12 (23.5)	1.0 (reference)
rs598126			
CC	58	15 (34.)	**2.2 (1.1**–**4.4)**
CT	119	37 (31.1)	1.4 (0.7–2.6)
TT	54	20 (27.8)	1.0 (reference)

**Hazard ratio for AD, adjusted for age, ethnicity, level of intellectual disability, BMI, and the presence of an *APOE ε*4 allele.

*Numbers vary because not all participants were genotyped for all SNPs.
